# Herpes Simplex Virus: Molecular Pathogenesis, Type-Specific Disease Burden, and Advances in Prevention and Treatment

**DOI:** 10.3390/v18101116

**Published:** 2026-10-09

**Authors:** Chenhao Jiang, Jianhui Nie, Li Zhang

**Affiliations:** 1 Division of HIV/AIDS and Sex-Transmitted Virus Vaccines, Institute for Biological Product Control, National Institutes for Food and Drug Control, Beijing 102629, China; 2 National Vaccine and Serum Institute (NVSI), Beijing 101111, China

**Keywords:** herpes simplex virus, HSV-1, HSV-2, immune evasion, latency, genital herpes, keratitis, vaccine, antiviral therapy

## Abstract

Herpes simplex virus (HSV) is a highly prevalent neurotropic DNA virus that causes orolabial herpes, genital herpes, keratitis, neonatal infection, meningitis and encephalitis. HSV-1 and HSV-2 share epithelial entry, lytic replication, immune evasion, lifelong sensory-ganglion latency and periodic reactivation, yet they differ in typical transmission routes, anatomical recurrence and public-health burden. Rather than mechanically separating both viruses throughout, this review emphasizes shared HSV biology and highlights type-specific differences where they affect clinical interpretation or intervention design. We connect glycoprotein-mediated entry, receptor use, axonal transport, immune evasion and latency with mucocutaneous, ocular, neurologic, neonatal and genital disease. We then synthesize lessons from failed glycoprotein-D vaccine trials, multivalent and mRNA platforms, helicase–primase inhibitors, monoclonal antibodies, RNA-based approaches, genome editing and latency-directed strategies. A mechanism-guided HSV strategy should reduce acquisition, shedding and recurrence, prevent ocular, neurologic and neonatal complications, manage antiviral resistance and ultimately address latent infection.

Herpes simplex virus (HSV) belongs to the Alphaherpesvirinae subfamily of the family Herpesviridae and comprises two closely related human serotypes, HSV-1 and HSV-2. HSV infection is widespread worldwide: recent estimates indicate that approximately 3.8 billion people younger than 50 years were infected with HSV-1 and approximately 520 million people aged 15–49 years were infected with HSV-2 in 2020 [1]. HSV-1 is commonly acquired through oral contact and is classically associated with orolabial disease, whereas HSV-2 is transmitted predominantly through sexual contact and remains the major cause of recurrent genital herpes. HSV is neurotropic: after primary epithelial infection it travels retrogradely along axons to sensory ganglia, where it establishes lifelong latency and can subsequently reactivate and return to peripheral tissues [2,3,4]. Most initial infections are asymptomatic or mild [5], but both types can cause severe disease. HSV-1 is the leading cause of sporadic viral encephalitis; HSV-2 is more often associated with aseptic meningitis and recurrent genital disease. Importantly, neonatal herpes can be caused by either type, and global modelling indicates that HSV-1 accounts for approximately one third of neonatal infections [6]. This review is organized around the mechanistic chain from viral entry and immune evasion to latency, disease burden, vaccines and therapeutics. We discuss HSV-1/HSV-2 differences when they alter tissue tropism, recurrence, transmission, neonatal risk or trial endpoints, while keeping the overall focus on HSV as a shared biological and translational problem.

## 1. Structure and Life Cycle of HSV

### 1.1. Structure of the Virion

**Figure 1:**
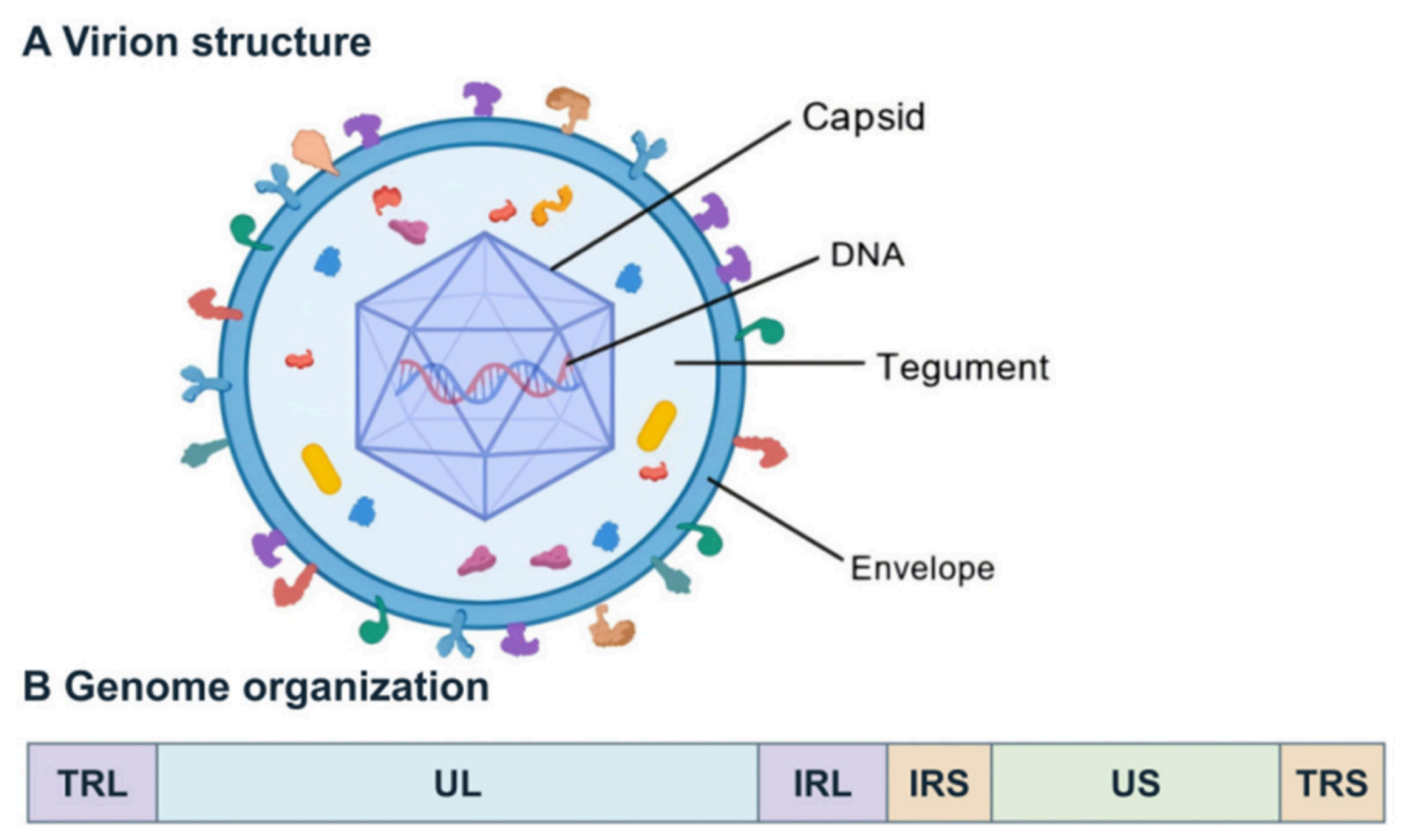
HSV virion structure and genome organization. (**A**) The virion contains a double-stranded DNA genome within an icosahedral capsid, a protein-rich tegument and a lipid envelope bearing glycoproteins. (**B**) The unique long (UL) and unique short (US) regions are flanked by terminal and internal inverted repeats (TRL/IRL and TRS/IRS). The genome is schematic and not drawn to scale.

HSV particles are spherical, with a diameter of approximately 150–200 nm, and consist of four main structural components (Figure 1): the innermost genome is composed of linear double-stranded DNA of about 152 kb; the genome is surrounded by an icosahedral capsid containing 162 capsomers; the capsid is embedded in a protein-rich tegument; and the outermost layer is a host-derived lipid envelope containing viral glycoproteins [7,8,9,10,11]. The HSV-1 capsid has a diameter of approximately 125 nm and is composed of hexameric and pentameric capsomers formed by the major capsid protein VP5, as well as trimeric proteins (VP19C and VP23) located between the capsomers. At one pentameric vertex, a dodecameric portal formed by UL6 mediates genome entry and packaging [12,13]. The tegument layer is rich in virus-encoded proteins such as VP16, UL36 and VP22, as well as selected host proteins and RNAs. The envelope contains at least 12 glycoproteins, including gB, gC, gD, gE, gG, gH, gI, gJ, gK, gL, gM and gN (Table 1). Among these, gD, gB and the gH/gL complex constitute the core cell attachment and membrane-fusion machinery [14,15]. Classical studies suggest that the HSV genome encodes approximately 80 open reading frames, while recent sequencing and functional-genomics studies indicate that the coding capacity may be substantially broader, illustrating the complexity of HSV gene regulation [16]. Comprehensive descriptions of HSV virion architecture, genome organization and the lytic cascade are available in reference texts [17,18].

HSV genes are conventionally grouped by expression kinetics into immediate-early (IE or alpha), early (E or beta), and late (L or gamma) classes. The incoming tegument transactivator VP16 recruits host factors to activate IE transcription. IE proteins such as ICP4 and ICP0 then promote E-gene expression; E products include enzymes required for viral DNA synthesis. Late genes can be further separated into leaky-late (γ1) genes, whose expression is enhanced but not absolutely dependent on viral DNA replication, and true-late (γ2) genes, whose robust transcription requires DNA replication. These kinetic distinctions coordinate the ordered production of regulatory proteins, replication machinery and structural components [19].

### 1.2. Infection and Replication Cycle

**Figure 2:**
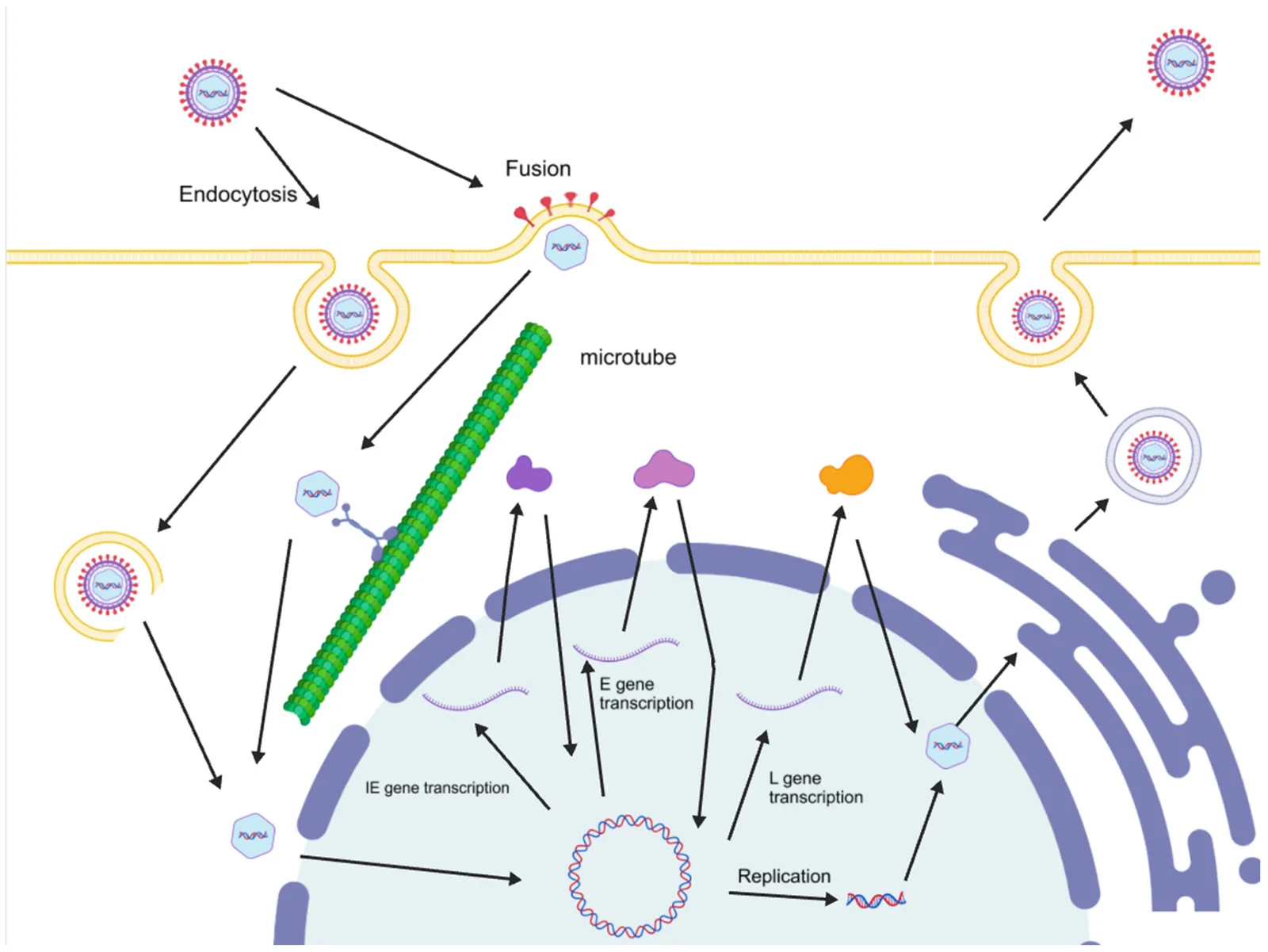
HSV entry, replication and egress. Attachment through gC/gB and receptor engagement by gD lead to gB–gH/gL-mediated fusion at the plasma membrane or, after endocytosis, an endosomal membrane. The capsid travels along microtubules to a nuclear pore and releases its genome into the nucleus. IE and early gene expression support DNA replication, followed by late gene expression, capsid assembly and genome packaging. Primary envelopment occurs at the inner nuclear membrane; fusion at the outer nuclear membrane releases the capsid into the cytoplasm. Tegument acquisition and secondary envelopment at trans-Golgi network-derived membranes precede vesicular transport and exocytosis.

The complete infection and replication cycle encompasses multiple stages, including viral attachment and entry, gene expression and DNA replication within the nucleus, capsid assembly and genome packaging, nuclear egress, secondary envelopment in cytoplasmic membranes and release of progeny virions (Figure 2). HSV infection begins when envelope glycoproteins bind host-cell receptors. Initially, positively charged gC and gB interact with negatively charged heparan sulfate proteoglycans on the cell surface, mediating attachment [20]. gD then binds high-affinity receptors such as nectin-1 or herpesvirus entry mediator (HVEM), triggering conformational changes in the gB-gH/gL fusion apparatus and release of the capsid into the cytoplasm. In some cell types HSV instead enters by receptor-mediated endocytosis, with subsequent fusion at the endosomal membrane. Once in the cytoplasm, the capsid translocates along microtubules to the nuclear pore, where the viral genome is released into the nucleus [21,22]. Immediate-early genes are expressed first, with ICP4 and ICP0 promoting early-gene expression. Early products, including the viral DNA polymerase, the single-stranded DNA-binding protein ICP8 and the helicase–primase complex UL5/UL8/UL52, drive genome replication. Empty capsids assembled in the nucleus package newly replicated DNA through the portal complex with the aid of packaging proteins such as UL6, UL15 and UL28 [23]. Mature nucleocapsids undergo primary envelopment at the inner nuclear membrane, de-envelopment at the outer nuclear membrane with release into the cytoplasm, followed by secondary envelopment at trans-Golgi network-derived membranes. Progeny virions are then transported within Golgi-derived vesicles and released by vesicle fusion at the plasma membrane into the extracellular space, enabling local spread, cell-to-cell transmission and neural dissemination [12].

### 1.3. Immune Evasion Strategies of HSV

HSV encodes a broad repertoire of proteins that blunt innate and adaptive antiviral responses. Phylogenetic analyses support codivergence of the HSV-1 lineage with the human lineage approximately six million years ago and a cross-species origin of HSV-2 through transmission from an ancestor of chimpanzees to an extinct human precursor approximately 1.6 million years ago; these dates are model-based estimates [24]. The long evolutionary history of primate simplex viruses provides context for their persistent lifestyle. Neuronal latency, together with immune-evasion mechanisms, allows HSV to persist despite immune control and presents challenges to effective vaccination. At the innate level, the virion host shutoff protein (vhs, encoded by UL41) degrades host mRNAs and dampens interferon (IFN)-stimulated responses, while the immediate-early protein ICP0, an E3 ubiquitin ligase, disrupts ND10/PML nuclear bodies and antagonizes type I IFN induction. ICP34.5 counteracts the PKR–eIF2α pathway to sustain viral protein synthesis and also antagonizes autophagy and STING-dependent DNA sensing [23,25]. HSV further impairs antigen presentation: ICP47 binds the transporter associated with antigen processing, blocking peptide loading onto MHC class I and reducing recognition by CD8+ T cells [26]. In addition, the gE/gI complex functions as an Fc receptor and gC binds complement component C3b, allowing the virus to blunt antibody- and complement-mediated effector functions [27,28].

Immune control requires complementary humoral and cellular mechanisms. Antibodies can inhibit entry and recruit complement or Fc-receptor-bearing effector cells, whereas natural killer (NK) cells and HSV-specific T cells help control infected cells and recurrent replication. Human studies of NK-cell dysfunction and tissue-resident CD8+ T cells support the importance of these cellular components [29,30]. Multivalent gC/gD/gE vaccines therefore aim to combine entry-blocking antibodies with antibodies that counter complement and Fc evasion; these activities complement, rather than replace, the cellular responses needed for sustained immune control.

### 1.4. Latency, Reactivation and Determinants of Type-Specific Tropism

A defining feature of HSV is lifelong latent infection in sensory neurons. After productive replication at epithelial or mucosal entry sites, nucleocapsids are transported retrogradely along axonal microtubules to neuronal cell bodies, where the genome circularizes and is maintained as a chromatinized episome [31]. During latency, lytic genes are largely silenced and packaged into repressive heterochromatin; the latency-associated transcript (LAT), viral miRNAs and host chromatin regulators contribute to repression of lytic gene expression and neuronal survival [32,33]. Reactivation may be triggered by ultraviolet exposure, fever, tissue injury, stress, hormonal changes or immunosuppression [34]. Both HSV-1 and HSV-2 can infect oral and genital sites, but their characteristic clinical patterns reflect differences in the frequency of reactivation at these sites. Both types use gD to engage nectin-1 and HVEM and use the gB-gH/gL fusion machinery, and clinical isolates can generally use both major gD receptors [35]; thus receptor choice alone does not establish strict oral or genital specificity. Instead, viral sequence variation, receptor accessibility, neuronal context, tegument-associated functions and local immune control collectively influence infection and recurrence. HSV-1 is more commonly associated with recurrent orolabial disease, whereas HSV-2 causes more frequent genital recurrences; prospective observations show that recurrence depends jointly on viral type and anatomical site [36].

Experimental genome substitutions provide evidence beyond receptor usage. Replacing HSV-2 LAT-region sequences with the corresponding HSV-1 sequences impaired genital reactivation in guinea pigs [37]. In a mouse ocular model, replacing the HSV-1 VP16 promoter/5′ untranslated region with its HSV-2 counterpart accelerated entry into the lytic programme in sensory neurons and enhanced responses to a reactivation stimulus [38]. VP16 is a tegument transactivator, but tegument delivered during primary infection should be distinguished from de novo VP16 expression during reactivation. These studies demonstrate contributions from type-specific genomic regulation and VP16-associated functions; they do not establish that VP16 protein-sequence differences alone determine human anatomical recurrence patterns. Genital HSV-1 nevertheless occurs increasingly, but recurs and sheds less often than genital HSV-2. In pregnancy, either type can be transmitted intrapartum, particularly after newly acquired maternal genital infection [6]. These outcomes emerge from interactions among viral genotype, entry route, ganglion biology and local innate, antibody and T-cell control. Because nucleoside analogues act on replicating virus, current therapy does not clear latent reservoirs.

## 2. Clinical Spectrum and Disease Burden of HSV

### 2.1. Mucocutaneous Herpes

The most common clinical manifestations of HSV are vesicles, erosions and ulcers on skin or mucosal surfaces. HSV-1 typically causes orolabial herpes and primary herpetic gingivostomatitis in children, whereas HSV-2 mainly causes recurrent genital and perianal herpes, with more frequent genital recurrence and shedding than genital HSV-1. This type-specific distinction is clinically useful but should not be treated as strict site specificity: either virus can infect oral or genital mucosa depending on exposure route [39,40]. From a prevention perspective, the key clinical issue is not only lesion location but also silent shedding, because asymptomatic mucosal replication sustains transmission and makes reduction of shedding an important endpoint for vaccines and antivirals.

### 2.2. Ocular and Central Nervous System Disease

Herpes simplex keratitis involves recurrent corneal epithelial infection and can be associated with stromal inflammation, corneal scarring and vision loss. Herpes simplex encephalitis (HSE), caused predominantly by HSV-1, is one of the most severe forms of sporadic viral encephalitis and carries substantial mortality and long-term neurological sequelae. Without acyclovir, mortality can exceed 70%. Autoimmune encephalitis may follow HSE after completion of antiviral therapy, particularly in children [41,42]. HSV-2 causes a smaller but non-trivial share of HSV encephalitis, chiefly in neonates and immunocompromised hosts, and more often presents as aseptic meningitis, including the recurrent (Mollaret) form; it is also the type most frequently implicated in neonatal central-nervous-system disease [41]. These syndromes illustrate why HSV prevention cannot be evaluated only by genital-lesion endpoints: ocular recurrence, neuronal invasion, central-nervous-system inflammation and post-infectious autoimmunity represent distinct clinical consequences of the same neurotropic life cycle.

### 2.3. Neonatal Herpes

Newborns are highly susceptible to HSV, acquired predominantly during passage through an infected birth canal. Both HSV-1 and HSV-2 cause neonatal herpes; modelling estimated approximately 14,000 incident cases worldwide in 2010 and attributed about one third to HSV-1 [6]. Neonatal HSV is classified as skin-eye-mouth disease, central nervous system disease, or disseminated multiorgan disease [43]. Disseminated and central nervous system forms have the poorest prognosis, and survivors may have long-term neurological sequelae despite antiviral treatment. Risk is driven especially by newly acquired maternal genital infection late in pregnancy, when maternal neutralizing antibody has not yet developed, rather than by viral type alone. Prevention therefore requires type-aware counselling, avoidance of new genital acquisition near term, recognition of active genital lesions or prodromal symptoms at delivery, suppressive antiviral therapy near term when indicated, and cesarean delivery when active genital disease is present [40].

### 2.4. Chronic Impact, HIV Interaction and Uncertain Associations

Recurrent HSV disease can cause pain, stigma, anxiety and impaired quality of life. HSV-2 has particular public-health importance because genital mucosal inflammation and recruitment of CCR5+ CD4+ T cells increase susceptibility to HIV acquisition [44,45]. HSV-1 contributes substantially to chronic morbidity through recurrent orolabial disease, keratitis and severe encephalitis. Some epidemiological studies have examined associations between genital HSV infection, high-risk human papillomavirus (HPV) infection and cervical neoplasia, but causality and independent contribution remain uncertain [46]. Similarly, HSV-1 DNA has been detected in human brain tissue, and an early case-control study reported an interaction between cerebral HSV-1 and APOE-epsilon4 in Alzheimer’s disease [47]; more recent registry studies have examined associations among herpesvirus diagnoses, antiviral exposure and dementia risk [48]. These observations justify mechanistic and prospective research, but they should be framed as unresolved associations rather than established causal indications for antiviral or vaccine intervention.

## 3. Progress in HSV Vaccine Research and Development

Despite the substantial disease burden caused by HSV, no vaccine for prevention or treatment of HSV infection has been FDA-approved; however, the engineered HSV-1-based oncolytic product talimogene laherparepvec (T-VEC) is FDA-approved for local treatment of specified melanoma lesions [49,50]. Approval for cancer treatment does not establish protection against HSV infection. Immune control of HSV depends on antibodies together with NK cells, CD4+ T cells and CD8+ T cells. Neutralizing and Fc-functional antibodies can limit extracellular virus and recruit effector activity, NK cells contribute to early antiviral defence, and tissue-resident T cells help contain recurrent infection at peripheral sites [29,30]. Vaccine development must therefore address mucosal entry and cell-to-cell spread, immune evasion affecting antibody and cellular responses, and persistence in neuronal reservoirs [51,52]. Most clinical development has focused on genital HSV-2 because of recurrence, shedding and HIV-related burden, but broader prevention should also consider genital HSV-1, ocular disease and neonatal exposure. Each platform should be matched to a defined outcome: preventing acquisition, reducing mucosal replication and shedding, preventing establishment of latency, or reducing reactivation in already infected individuals. Table 2 summarizes representative historical and contemporary platforms and distinguishes preclinical findings from clinical evaluation.

### 3.1. Live-Attenuated and Replication-Defective Vaccines

Live-attenuated and replication-defective HSV vaccines present multiple viral antigens, but their replication capacity and safety properties differ. The early glycoprotein-H-deleted disabled infectious single-cycle (DISC) platform required production on gH-complementing cells; complemented inoculum could infect cells, but progeny could not sustain spread in non-complementing tissue. A genetically disabled HSV-1 candidate protected guinea pigs against primary and recurrent HSV-2 disease [53]. The subsequent randomized therapeutic trial of a gH-deleted HSV-2 vaccine found acceptable safety but no clinical or virological benefit in immunocompetent adults with recurrent genital herpes [54]. These findings, placed in the context of the early HSV-vector literature [55], show that promising animal protection and restricted spread do not ensure human therapeutic efficacy.

Deletion of γ34.5, encoding ICP34.5, provided another early attenuation strategy. Studies of HSV1716 in the 1990s established marked attenuation in mouse models and motivated investigation as a vaccine or vector backbone [56]. ICP34.5 deletion restricts replication in particular host contexts but does not make a virus universally replication defective. This attenuation principle subsequently contributed to oncolytic platforms, including T-VEC; cancer efficacy must not be interpreted as efficacy of an HSV vaccine. Earlier inactivated whole-virus approaches are of historical interest, but convincing controlled evidence of protection remains insufficient and these approaches are not a basis for assuming effective immune control [51,57].

A later replication-defective platform, dl5-29/HSV529, lacks UL5 and UL29. Its randomized phase I trial demonstrated safety and neutralizing-antibody responses with modest CD4+ T-cell responses in HSV-seronegative vaccine recipients; protective efficacy was not established [58]. Broad antigen presentation is attractive, but safety, neuronal persistence and performance in immunocompromised recipients require careful evaluation [51,59]. An HSV-2 mutant deleted in gD (ΔgD-2), produced on gD-1-complementing cells and restricted to a single infectious cycle, protected mice against lethal vaginal and skin challenge and prevented detectable ganglionic latency [60]. Protection was transferable with immune serum and depended on FcγR-mediated effector function rather than classical neutralization [61]. These experiments show why antigen selection and assays of Fc-effector activity deserve evaluation alongside serum neutralization. They do not establish that deleting gD will confer the same advantage in humans.

### 3.2. Subunit Vaccines

Subunit vaccines have generally focused on entry glycoproteins, particularly gD and gB. The best-known candidate was the GSK recombinant gD2 vaccine formulated with alum and monophosphoryl lipid A. Two randomized efficacy trials reported protection against genital herpes disease in women who were seronegative for both HSV-1 and HSV-2 and whose regular partners had a history of genital herpes, but not in men or HSV-1-seropositive women [57,62]. The subsequent Herpevac Trial for Women enrolled women seronegative for both types without requiring a known infected partner. It protected against HSV-1 genital disease and infection, but not against HSV-2 disease or infection [63]. Vaccine-recipient sera neutralized HSV-1 more efficiently than HSV-2, although this observation does not fully explain the difference in clinical efficacy [64].

These outcomes show that a potent serum response to one entry antigen does not ensure broad immune control. gD antibodies can block receptor engagement, but they do not cover every mechanism of attachment, fusion and cell-to-cell spread; baseline serostatus and mucosal immunity can also alter effectiveness. A gD-only vaccine does not directly target the full immune-evasion repertoire of challenge virus, and serum neutralization does not establish durable tissue-resident T-cell responses or eliminate genomes already latent in neurons. Multivalent gC/gD/gE designs address complementary functions: gD induces entry-blocking antibodies; gC immunization aims to inhibit C3b binding and complement evasion; and gE immunization targets gE/gI-mediated Fc evasion. Animal studies support improved protection through these combined mechanisms [28,65], but their translation to human efficacy remains unproven. Vaccine-induced protection in humans remains a goal that will probably require coordinated antibody, Fc-effector and tissue-resident T-cell immunity.

### 3.3. Nucleic Acid Vaccines

Nucleic acid vaccines include DNA vaccines and mRNA vaccines. DNA vaccines, such as the recently reported SL-V20 multivalent DNA vaccine, utilize plasmids to simultaneously express multiple HSV-2 antigens and immune stimulatory molecules, reducing genital tract lesions and promoting virus clearance in mouse models [66]. BioNTech’s BNT163 is a trivalent mRNA vaccine encapsulated in lipid nanoparticles that targets HSV gC2/gD2/gE2. Mouse experiments showed strong protection against fatal infection, genital lesions and ganglionic invasion [67,68]. Compared with traditional protein vaccines, mRNA platforms can express multiple conformational antigens in situ and may induce broader cellular and humoral immunity in animal models. The key translational question is whether these responses reduce human acquisition, shedding and ganglionic seeding, rather than only preventing lesions in challenge models.

### 3.4. Other Vaccine Platforms

In addition to conventional prophylactic and therapeutic vaccines, HSV vectors have been engineered as oncolytic immunotherapies. Talimogene laherparepvec (T-VEC) is an attenuated HSV-1 lacking ICP34.5 and ICP47 and expressing GM-CSF; in the phase III OPTiM trial it improved durable response rate in advanced melanoma and subsequently became the first approved oncolytic HSV therapy [69]. A further example is G47Δ, a third-generation, triple-mutated oncolytic HSV-1 that met its primary endpoint in a single-arm phase 2 trial in residual or recurrent glioblastoma and became the first oncolytic virus product approved in Japan; serial biopsies showed progressively increasing tumour infiltration by CD4+ and CD8+ lymphocytes [70]. Together, these programmes show that engineered HSV can be administered repeatedly into human tissue, can replicate locally under attenuating mutations and can recruit T cells in situ, properties that are directly relevant to therapeutic vaccine design. This experience demonstrates that HSV attenuation can be combined with local replication, antigen release and immune stimulation, but antitumour efficacy should not be conflated with protection against wild-type HSV acquisition. For preventive vaccines, other platforms include viral vectors, virus-like particles and computationally selected antigens designed to broaden B- and T-cell responses [71,72,73]. Overall, next-generation design should connect antigen selection to defined mechanisms of entry or immune evasion and should test mucosal immunity, ganglionic seeding, recurrence and shedding rather than serum neutralization alone.

### 3.5. Trial Design, Endpoints and Current Development Status

Vaccine goals also shape design and regulatory endpoints. For prophylactic vaccines, prevention of acquisition, prevention of clinically apparent disease, reduction of mucosal shedding and prevention of ganglionic seeding are distinct outcomes. For therapeutic vaccines, which aim to reduce recurrences in already infected individuals, T-cell immunity at the site of reactivation is thought to be critical, and trials typically adopt reductions in lesion rate and viral shedding as endpoints [74,75]. Because HSV-1 and HSV-2 differ in typical clinical outcomes, trials should predefine whether oral, ocular, genital, neurologic or neonatal endpoints are being evaluated rather than treating all HSV outcomes as interchangeable. Serostatus, sex, anatomical site and recurrence frequency should be stratified because each can influence observed efficacy.

Reflecting these lessons, Table 2 includes historical single-cycle and neuroattenuated platforms alongside protein, peptide, DNA and mRNA candidates. Trivalent gC2/gD2/gE2 mRNA vaccination has produced strong protection against HSV challenge in mice [67,68], but prevention of lesions or detectable ganglionic seeding in animals does not establish prevention of acquisition or durable immune control in humans. As of 3 October 2026, no vaccine for prevention or treatment of HSV infection is FDA-approved [49]. Trial completion and immune-response measurements should not be interpreted as proof of protective efficacy.

## 4. Antiviral Therapy and Drug-Development Strategies

Systemic acyclovir and valacyclovir provide substantial clinical benefit in HSV disease: episodic treatment improves symptomatic episodes, and suppressive treatment reduces recurrent genital disease and viral shedding. Valacyclovir is an orally absorbed prodrug of acyclovir, and long-term suppressive use has a well-established safety record [40]. Their success in controlling disease should be distinguished from their inability to eradicate neuronal latency. Clinical urgency, route and duration differ among mucocutaneous, ocular, neonatal and central nervous system infections. Topical treatments have limited benefit for recurrent mucocutaneous disease, although this should not be generalized to specialist-directed ophthalmic treatment of epithelial keratitis [40].

The following sections distinguish inhibition of new entry events from inhibition of intracellular replication, and separate cell-culture or animal observations from human clinical evidence. Entry-blocking agents are particularly relevant to prophylaxis; effects on subsequent rounds of spread may also be useful, but therapeutic benefit requires direct clinical testing. Genome editing and durable epigenetic repression remain experimental approaches to latent reservoirs. Thus, established disease control, prevention of transmission and elimination of latency are separate therapeutic endpoints.

### 4.1. Nucleoside Analogues and DNA Polymerase Inhibitors

Nucleoside analogues are the cornerstone of HSV treatment. Acyclovir and penciclovir, the active drugs delivered directly or through the prodrugs valacyclovir and famciclovir, respectively, require initial phosphorylation by viral thymidine kinase (TK) followed by cellular phosphorylation and inhibit viral DNA polymerase [76,77]. Systemic acyclovir is essential for severe HSV syndromes, while oral acyclovir or valacyclovir is used for episodic and suppressive treatment of genital herpes. Suppressive therapy reduces the frequency of recurrent genital herpes by approximately 70–80% in patients with frequent recurrences [40].

Transmission reduction is real but incomplete and population-dependent. In a randomized trial of immunocompetent heterosexual HSV-2-discordant couples, daily valacyclovir reduced overall partner acquisition from 3.6% to 1.9% over eight months (hazard ratio 0.52), alongside counselling and access to condoms [78]. This finding does not establish elimination of transmission or a population-wide effect, and it should not be extrapolated to genital HSV-1 without direct evidence. Neither acyclovir nor valacyclovir removes latent neuronal genomes, and recurrences may resume after suppressive therapy is stopped.

Resistance through TK or polymerase mutations occurs particularly in immunocompromised patients. Foscarnet inhibits viral DNA polymerase without TK-dependent activation and is an established option for acyclovir-resistant genital infection [40]. Cidofovir also bypasses viral TK, but toxicity and administration constraints limit its use; brincidofovir should not be presented as routine established HSV treatment [77]. These alternatives inhibit replication rather than eliminate latency. Their value in resistant disease must be assessed separately from evidence for prevention of sexual transmission.

### 4.2. Other Small Molecule Drugs and Host-Targeted Drugs

Entry inhibition aims to prevent new infection events by interfering with attachment or fusion. Docosanol 10% cream is a topical treatment for recurrent herpes labialis, and cell-culture experiments support inhibition of viral entry [79]. Its clinical effect is modest: in a randomized placebo-controlled trial initiated at the prodrome or erythema stage, median healing time was 4.1 days, approximately 18 h shorter than with placebo [80]. This does not establish prevention of future recurrences, transmission or latent infection. Topical therapy for genital herpes offers little clinical benefit and is discouraged in current guidance [40].

Sulfated attachment inhibitors and nanomaterials remain experimental. For example, silver nanoparticles inhibited HSV-1 attachment and replication-associated endpoints in cell-culture experiments [81]. Such findings do not demonstrate benefit in patients, and mucosal safety, achievable exposure and efficacy require clinical testing. Preventing entry into uninfected cells should not be conflated with inhibiting viral DNA replication within already infected cells or targeting a latent neuronal genome.

### 4.3. Protein and Peptide Therapy

Proteins and peptides offer experimental ways to interfere with HSV attachment and infection. Lactoferrin and derived peptides have shown anti-HSV activity in experimental systems, supporting investigation as mucosal or topical candidates [82]. Such observations do not establish clinical efficacy for treatment of established HSV disease. Host-directed cytokine strategies likewise require evidence of benefit and tolerability; they should not be presented as substitutes for standard antiviral therapy. For these approaches, local delivery, tissue toxicity and the distinction between prophylactic exposure and treatment after infection remain central translational questions.

### 4.4. Monoclonal Antibody Therapy

Virus-specific monoclonal antibodies are being investigated for prophylaxis and treatment. Experimental studies of the gD-directed antibody 4A3 support entry inhibition and protection in preclinical models [83]. Studies of the gB-directed antibodies HDIT101 and HDIT102 identified complementary binding and antiviral activity, including effects on gB trafficking and improved protection with the combination in experimental systems [84]. The non-neutralizing antibody BMPC-23 engages Fc-gamma receptor IV and enhances antibody-dependent cellular cytotoxicity (ADCC), protecting mice against HSV disease [85]. These results support testing combinations of entry blockade and Fc-mediated effector activity; they do not establish that the reported combinations prevent human transmission or eliminate latency. Clinical efficacy must be determined for each product, regimen and indication.

### 4.5. RNA-Based and Genome-Editing Therapies

Sequence-directed nucleic-acid approaches include RNA interference, miRNA modulation and genome editing. Small interfering RNAs targeting essential HSV transcripts can suppress lytic replication in cultured cells, but transient activity and delivery to infected mucosa and neurons remain major barriers [86]. Endogenous miRNAs also participate in HSV infection and host antiviral regulation. HSV encodes multiple miRNAs, and host miRNAs can alter viral replication; for example, ICP4-induced miR-101 downregulates the host RNA-binding protein GRSF1 and thereby attenuates HSV-1 replication [87]. Artificial use of miRNA mimics or antagonists may therefore modulate latency and reactivation, but specificity, durability and delivery remain unresolved. CRISPR-Cas9 delivered by a single AAV9 vector reduced HSV-1 DNA/RNA expression and reactivation-associated shedding in a latent rabbit keratitis model [88]. In parallel, HSV-specific meganucleases delivered with selected AAV serotypes eliminated more than 90% of latent HSV DNA from some mouse ganglia in 2020 [89]; a subsequent study reported up to approximately 97% reduction in ganglionic viral load and decreased reactivation-associated shedding in oral and genital mouse models [90]. These results establish proof of concept for reservoir reduction rather than clinical cure. Translation will require efficient and sufficiently uniform transduction of trigeminal and sacral neuronal subtypes, dosing that reaches anatomically dispersed ganglia, durable nuclease expression without neurotoxicity, scalable administration, and assays that can measure residual latent genomes. CRISPR-Cas9 additionally raises risks of host-genome off-target cleavage, unintended consequences of on-target cleavage and neuronal injury, immune responses to Cas proteins and AAV, vector biodistribution outside target ganglia, and selection of nuclease-resistant viral variants. Multiplex targeting may reduce viral escape but increases payload and safety complexity.

### 4.6. Drug Resistance Mechanisms and Surveillance

Because the activity of nucleoside analogues depends on viral thymidine kinase (TK) and DNA polymerase, mutations in the corresponding genes—UL23 (TK) and UL30 (DNA polymerase)—are the molecular basis of acquired resistance [91,92]. Most clinical resistance arises from TK alterations, which range from complete loss of activity (TK-negative) to reduced or substrate-altered activity (TK-low or TK-altered); these genotypes differ in clinical significance, because TK-negative strains are cross-resistant to all TK-dependent drugs—acyclovir and its prodrug valacyclovir, as well as penciclovir and its prodrug famciclovir—yet usually remain susceptible to foscarnet and cidofovir, which do not require TK activation. It follows that an acyclovir-resistant, TK-negative isolate cannot be rescued simply by switching to valacyclovir or famciclovir, since these share the same activation step. Polymerase (UL30) mutations are less common and may additionally confer resistance to foscarnet. The prevalence of resistance is low in immunocompetent individuals (well under 1%) but rises substantially—commonly several percent and occasionally higher—in immunocompromised hosts such as hematopoietic stem-cell and solid-organ transplant recipients, reflecting prolonged viral replication under drug pressure together with impaired immune clearance. These differences argue for genotypic and phenotypic resistance testing, and for ongoing resistance surveillance, particularly in transplant and HIV-positive populations.

Many TK-deficient resistant mutants carry fitness costs, including impaired replication in neurons and reduced reactivation, which helps explain why difficult within-host resistant infections do not necessarily translate into widespread community dissemination [91,93]. However, fitness depends on the mutation and viral background. TK-negative mutants can establish latency in experimental models, and reactivation of a clinically derived TK-deficient resistant strain has been reported [93,94]. Resistant isolates should therefore not be described as universally unable to persist or transmit. Selection of resistance during treatment does not demonstrate wholesale replacement of the pre-existing latent reservoir; surveillance should remain focused on prolonged or refractory infection, especially in immunocompromised patients.

### 4.7. Novel Targets and Combination Strategies

The viral helicase–primase complex (UL5/UL8/UL52) is a clinically investigated target distinct from DNA polymerase. In randomized human studies, pritelivir reduced genital HSV-2 shedding and was compared with valacyclovir [95,96]; these trials provide clinical evidence for replication suppression rather than eradication of latency or proven prevention of transmission. Helicase–primase inhibition does not require viral TK, providing a rationale for activity against TK-deficient, acyclovir-resistant isolates. Experimental agents with improved tissue exposure and approaches targeting capsid assembly or egress require their own pharmacological and clinical validation.

Combination and host-directed strategies aim to raise the genetic barrier to resistance and ultimately address latency. Co-administration of a nucleoside analogue with a helicase–primase inhibitor can suppress lytic replication through independent targets, while immunomodulatory combinations seek to strengthen host control. Latency-directed strategies have distinct goals. A true ‘shock-and-kill’ approach would deliberately induce lytic gene expression from quiescent episomes and then eliminate reactivated virus or infected neurons with antivirals and immune effectors. However, the cited LSD1 study does not demonstrate such a latency-reversing intervention: inhibition of the histone demethylase LSD1 blocked HSV immediate-early transcription, lytic replication and reactivation [97], making LSD1 inhibition conceptually closer to suppressing reactivation than to ‘shock’. Shock-and-kill also risks provoking lesions, neuronal injury or CNS disease; asynchronous and incomplete reactivation leaves residual episomes, while conventional antivirals stop DNA replication without necessarily eliminating the neuron that carries the genome. ‘Block-and-lock’ instead seeks durable epigenetic silencing of the latent episome. Its principal challenges are achieving lifelong repression across heterogeneous neuronal reservoirs, avoiding disruption of host chromatin and neuronal gene expression, and demonstrating that stress or immunosuppression cannot reverse the locked state. For both strategies, sensory-ganglion penetration is constrained by anatomical dispersion, neuronal subtype tropism, blood-nerve barriers and vector dose; animal latency models incompletely reproduce spontaneous human recurrence and shedding. These delivery, safety, reversibility, manufacturing and long-term follow-up requirements currently keep both concepts largely preclinical.

### 4.8. Natural Products and Traditional Medicines

A range of natural products has shown anti-HSV activity in vitro and in animal models and may provide scaffolds for adjunctive therapy. Flavonoids and triterpenoids such as baicalin and glycyrrhizic acid, for example, have been reported to inhibit HSV replication and to modulate virus-induced inflammation [98]. Clinical evidence remains limited; however, their mechanisms of action, potency, and bioavailability require further study before firm therapeutic claims can be made.

## 5. Knowledge Gaps and Future Directions

Future HSV research should change to mechanism-linked prioritization. First, protective immunity needs better definition at mucosal and neuronal interfaces. Serum neutralization remains useful but insufficient; studies should measure complement-restoring antibodies, Fc-effector activity, local CD4+ and CD8+ T-cell responses, tissue-resident memory, mucosal shedding and ganglionic seeding. These immune correlates should be interpreted by viral type, baseline serostatus, anatomical site and sex.

Second, antiviral development should distinguish suppression of lytic replication from control of latency. Polymerase and helicase–primase inhibitors suppress replication, while antibodies and entry inhibitors may restrict new infection events or spread. RNA interference generally targets expressed viral transcripts and should not itself be equated with elimination of latent DNA. Genome-editing and epigenetic approaches seek reservoir reduction or durable repression, but require delivery systems that reach neuronal reservoirs safely and reproducibly, limit off-target injury, avoid neurotoxicity and prevent viral escape. Demonstrated reduction of shedding must also be distinguished from direct evidence of reduced transmission.

Third, clinical translation should use endpoints matched to clinical need. Genital HSV-2 trials should emphasize recurrence and quantitative shedding; genital HSV-1 studies may require different follow-up because recurrence is often less frequent; ocular disease requires recurrence, inflammation and visual outcomes; and neonatal prevention depends on maternal acquisition, serostatus and peripartum exposure. A unified HSV strategy will therefore need type-aware trial design without fragmenting the review into parallel HSV-1 and HSV-2 narratives.

## 6. Conclusions

Current nucleoside analogues provide effective clinical treatment and suppression of recurrent HSV disease, but they target replicating virus and do not eradicate latent infection. Helicase–primase inhibitors act independently of viral thymidine kinase and may retain activity against TK-deficient, acyclovir-resistant isolates [95,96], but even potent replication inhibitors cannot remove chromatinized latent episomes from neurons. A realistic therapeutic roadmap therefore requires layered interventions: suppressive antivirals for lytic replication, resistance-guided alternatives for immunocompromised patients, antibodies to limit entry or spread and RNA-based agents to suppress viral transcripts, and latency-directed strategies to durably silence or eliminate viral genomes. AAV-delivered meganucleases have reduced latent ganglionic HSV loads and reactivation-associated shedding in mice [89,90], while CRISPR-Cas9 has shown activity in a latent rabbit ocular model [88]; neither approach has yet established safety, delivery coverage or durable efficacy in humans. Vaccine trials likewise should distinguish acquisition, disease, ganglionic seeding and shedding and should stratify participants by viral type, baseline serostatus, sex, immune status and anatomical syndrome. The central lesson is that HSV control is unlikely to follow from a single antigen or a single replication inhibitor. Progress will depend on mechanism-linked combinations that counter entry and immune evasion, generate durable mucosal and tissue-resident immunity, manage resistance and safely address the latent reservoir.

## Figures and Tables

**Table 1:** Envelope glycoproteins of HSV.

Glycoprotein	Encoding Gene	Function
gB	*UL27*	Type I membrane protein, the viral fusion protein (fusogen), binds to heparan sulfate on the cell surface and participates in cell entry
gC	*UL44*	Type I membrane protein; binds heparan sulfate for attachment and C3b to inhibit complement-mediated antiviral activity
gD	*US6*	Type I membrane protein; binds to cell surface receptors; involved in cell adhesion
gE	*US8*	Type I membrane protein; forms a complex with gI to constitute FcR
gG	*US4*	Type I membrane protein; virus chemokine binding protein
gH	*UL22*	Type I membrane protein; forms the gH/gL complex that regulates membrane fusion and participates in cell entry
gI	*US7*	Type I membrane protein; forms a complex with gE to constitute FcR
gJ	*US5*	Type I membrane protein; associated with virus replication; inhibits apoptosis
gK	*UL53*	Multipass membrane protein; contributes to membrane fusion, cytoplasmic envelopment and egress
gL	*UL1*	Forming a complex with gH and participating in cell entry
gM	*UL10*	Type III membrane protein; eight transmembrane domains; forms a complex with gN; ensures the correct acquisition of the final envelope on the TGN of the viral nucleocapsid
gN	*UL49.5*	Type I membrane protein; forms a complex with gM

**Table 2:** Representative HSV vaccine platforms and historical or registered clinical evidence.

Class	Developer	Name	Identifier	Antigen/Adjuvant	Classification	Clinical Stage
Subunit vaccine	GSK	GSK208141	NCT00698893; NCT00697567; NCT00224471	gD	Prophylactic	Completed (I/II/III)
			NCT00698490	gD2	Prophylactic	Completed (II)
			NCT00699764	gD2 + MPL	Prophylactic	Completed (III)
			NCT00224484	gD2 + AS04	Prophylactic	Completed (III)
		GSK3943104A	NCT05298254	/	Therapeutic	Completed (I/II)
	Genocea	GEN-003	NCT01667341; NCT02114060; NCT02300142; NCT02515175; NCT03146403	gD2ΔTMR + ICP4.2 + Matrix-M2	Therapeutic	II (Completed/partial terminated)
	Novartis	gB2/gD2 + MF59	/	gB2 + gD2 + MF59	Prophylactic	III (Completed)
	BioNTech	gC2/gD2/gE2 + CpG/alum	/	gC2 + gD2 + gE2 + CpG + alum	Prophylactic	Preclinical/early stage
Synthetic peptide vaccine	Agenus	HerpV	NCT01687595; NCT0231049	HSP70-peptide complex; QS-21	Therapeutic	II (Completed)
DNA vaccine	PowderMed	pPJV7630	NCT00310271	Ubiquitinated and unmodified gD2	Therapeutic	I (Completed)
	Vical	VCL-HB01	NCT02837575	gD2 + UL46 + Vaxfectin	Therapeutic	II (Completed)
	Anteris	COR-1	/	gD2 codon optimization DNA	Therapeutic	Stop after phase IIa
mRNA vaccine	GSK	GSK-4108771A	NCT04762511	gE/gI (SAM)	Prophylactic	I (Terminated)
	BioNTech	BNT163	NCT05432583	gC2/gD2/gE2	Prophylactic	I (Active, not recruiting)
	Moderna	mRNA-1608	NCT06033261	/	Therapeutic	I/II (Completed)
Live attenuated and replication-defective vaccine	Sanofi Pasteur	HSV15	NCT04222985	HSV15 ± G103/GLA-SE	Therapeutic	I (Terminated)
		HSV529	NCT01915212; NCT02571166	UL5, UL29 deletion	Prophylactic	I (Completed)
	Rational Vaccines	RVx-201	/	ICP0 deletion	Therapeutic	/
		RVx-2001	/	HSV2	Prophylactic	/
		RVx-1001	/	HSV1	Prophylactic	/
	X-Vax	Delta gD-2	/	gD deletion	Prophylactic	/
	AuRx	ICP10ΔPK	/	ICP10ΔPK	Therapeutic	Stop after phase IIa

“/” represents undisclosed information. All trial information was obtained from https://classic.clinicaltrials.gov.

## Data Availability

No new data were created or analyzed in this study. Data sharing is not applicable to this article.
